# Proximal femur structural geometry changes during and following lactation

**DOI:** 10.1016/j.bone.2010.11.016

**Published:** 2011-04-01

**Authors:** M.A. Laskey, R.I. Price, B.C.C. Khoo, A. Prentice

**Affiliations:** a MRC Human Nutrition Research, Cambridge, UK; b Medical Technology and Physics, Sir Charles Gairdner Hospital, Perth, Australia; c School of Surgery, University of Western Australia, Perth, Australia

**Keywords:** Lactation, Femur structural geometry, Body weight, Calcium

## Abstract

Human lactation is associated with transient decreases in bone mineral density (BMD). Bone strength is related to both mass and structural geometry. This study investigated longitudinal changes of hip bone strength during lactation using hip structural analysis (HSA), which determines hip structural geometry (including areal BMD, BMDa) from dual-energy X-ray absorptiometry scans (DXA).

Forty-eight lactating women were studied longitudinally at the proximal femur using DXA at approximately 2 weeks postpartum, peak-lactation and post-lactation. Nonpregnant, nonlactating women (NPNL, *n* = 23) were studied concurrently at baseline and after 1 year. Hip scans were analysed using HSA at the narrow neck, intertrochanter and proximal shaft.

No significant change (> 0.05) was observed in NPNL women for any measurement. In contrast, for lactating women BMDa decreased significantly from 2 weeks postpartum to peak-lactation at narrow neck (−2.8%), intertrochanter (−3.2%) and shaft (−1.4%). Cross-sectional area (CSA) decreased at narrow neck (−3.4%) and intertrochanter (−2.7%). There were no significant changes in bone width. Section modulus decreased at intertrochanter (−2.1%). At shaft, cortical thickness decreased (−1.7%) and buckling ratio increased (2.3%). By post-lactation, measurements were not significantly different from 2 weeks postpartum except for decrements in BMDa (−1.1%) and CSA (− 1.2%) at the shaft. During the study, lactating women lost 5% of their body weight. Adjusting for weight changes decreased the magnitude and significance of HSA changes at peak-lactation and by post-lactation there were no significant differences from 2 weeks postpartum. Calcium intake was not a significant predictor of changes in HSA variables.

In conclusion, lactation is associated with significant but transient changes in hip BMD and structural geometry. Changes in body weight but not calcium intake were associated with these changes. These small changes at the hip during lactation occurred mainly at internal surfaces and had minimal impact on bending or compressive strength.

## Research highlights

►Lactation is associated with transient changes in hip BMD and structural geometry. ►Body weight, but not calcium intake, is associated with changes in bone. ►Lactation associated changes occur mainly at endosteal surfaces of hip. ►Changes have minimal impact on hip bending and compressive strength.

## Introduction

A lactating mother secretes about 200–300 mg/day of calcium into her breast milk [1]. This extra demand for calcium represents a considerable proportion of the calcium intake for many lactating women [2]. Dual-energy X-ray absorptiometry (DXA) studies have demonstrated that during the first 3–6 months of lactation, there are temporary decreases of bone mineral (reported as areal bone mineral density [BMDa] or bone area adjusted bone mineral content [BA-adj BMC]) at the total hip (–1% to −4%) and femoral neck (–2% to –7%) [2–9]. The bone mineral changes during lactation are greater and more rapid than the average annual bone mineral loss of about 1–3% experienced by postmenopausal women [2,10]. This release of calcium from the maternal skeleton may provide some of the extra calcium required for breast milk production. There has been concern that this decrease in bone mineral could lead to reductions in the bone strength of lactating mothers and make them more prone to fracture in later life. Although uncommon, fractures during lactation are well documented [11,12]. However, in one of these studies some women were known to have low bone density and/or other risk factors for osteoporosis [11]. In addition, retrospective studies investigating the relationship between parity and/or lactation history and fracture risk and bone mineral status are conflicting. Several studies show no relationship [13,14]. Other studies report an increased risk of lower bone mineral [15]. However, many studies report an improved bone status [16] or a reduced fracture risk as a result of breast feeding or high parity [17–21].

Bone strength is related not only to bone mass but also to bone structural geometry. Bone structural geometry is the architectural arrangement of bone tissue around the bone axis along, or about which it is loaded. Hence, if there are compensating changes to bone structural geometry it is possible for bone mineral mass to decrease with no, or minimal compromise to mechanical strength [22,23].

It is now possible to use biomechanical engineering principles to investigate bone geometry from projected 2-D images of the hip generated from DXA scans using the Hip Structural Analysis (HSA) method [24,25]. This uses raw spatial and mineral mass DXA information from the proximal femur to compute structural geometrical variables at three specific sites: the narrow neck, intertrochanteric and proximal shaft regions.

The aims of this study were to investigate longitudinal changes in hip structural geometry during and after lactation, in comparison to 2 weeks postpartum, in a group of British women to (i) determine if the observed decreases in bone mineral mass during lactation had an influence on bone strength, (ii) compare the results with nonpregnant, nonlactating (NPNL) women measured concurrently and (iii) consider the possible associations of body weight changes and calcium intake on changes in variables measured using HSA.

## Methods

### Study design and subjects

The lactating and NPNL women were a subset of women who were participating in a larger, longitudinal study designed to investigate the influence of lactation on bone. Details of these women have been reported previously [2,4]. This paper includes data from 48 women who lactated for more than 3 months and 23 NPNL women studied concurrently. It also includes one extra NPNL and one lactating woman whose data were not available at the time the previous papers were completed. The inclusion of NPNL women in the study enabled consideration of the potential skeletal changes in women due to advancing age and also investigated possible shifts in DXA performance over the study period. Approval for the study was obtained from the Ethical Committee of the MRC Dunn Nutrition Unit (of which MRC Human Nutrition Research was formerly a part) and written informed consent was obtained from each participant.

Lactating mothers visited the Dunn Clinical Nutrition Centre, Cambridge, UK at approximately 2 weeks postpartum, and for repeat measurements at 3, 6 and 12 months postpartum. An additional visit was made 3 months after breast feeding had stopped for women who lactated for more than 9 months. Peak-lactation was defined as 3 months postpartum for the 13 mothers who breast-fed for 3–6 months and 6 months postpartum for the 35 mothers who breast-fed for more than 6 months. Post-lactation was defined as 1-year postpartum for the 25 women who lactated for less than 9 months and 3 months post-lactation for the 21 women who lactated for more than 9 months. Two women were unable to be measured post-lactation because they had become pregnant again. All but one of the women was amenorrheic at the time of their peak-lactation visit and all women had resumed menstruation at the time of their post-lactation visit. Measurements were performed on the following days postpartum, expressed as mean (standard deviation [SD], range): 2 weeks postpartum 17 (5, 10–42) days, peak-lactation 159 (42, 85–226) days, post-lactation 426 (131, 269–932) days. Results reported for lactating women are changes from 2 weeks postpartum to peak-lactation and from 2 weeks postpartum to post-lactation. Results reported for NPNL women are changes from baseline to 319 (67, 152–406) days after baseline.

### DXA and other measurements

**Fig. 1:**
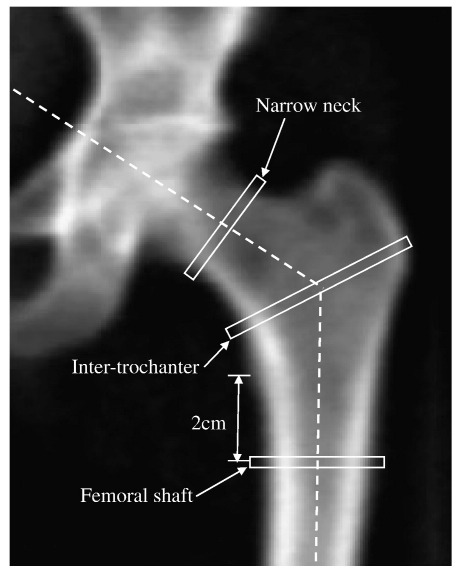
Hip image from Hologic DXA scanner showing locations of HSA narrow neck, intertrochanteric and femoral shaft regions of interest (ROI). Width of ROI is 3 mm.

Bone mineral measurements on the left hip were performed using DXA (QDR-1000W; Hologic Inc, Bedford, MA). Hip scans were analysed using the hip structural analysis (HSA) program (version 1) [26]. This is a computational algorithm applied to 2-D projected images of the hip generated from DXA scans, following conventional bone mineral analysis. The program uses the distribution of mineral mass in a line of pixels across the bone axis to compute cross-sectional structural geometry outcomes (e.g., CSA) in cut planes traversing the bone at three specific locations. These locations are: the narrow neck (region of interest [ROI] of 3-mm width, at the narrowest portion of femoral neck), intertrochanter (ROI of 3-mm width, along the bisector of the neck-shaft angle) and proximal shaft (ROI of 3-mm width, through the femoral shaft and located 2 cm distal to the user-defined midpoint of the lesser trochanter) as shown in Fig. 1. The narrow neck region approximates to the femoral neck region reported for conventional DXA scans, though lying proximal to it for Hologic machines. There are no conventional DXA-equivalent regions for intertrochanteric and shaft HSA regions.

Areal bone mineral density (BMDa), cross-sectional area of mineralized cortical/trabecular bone (CSA, an index of resistance to axial loading, closely related to bone mineral content), outer diameter (bone width) and section modulus (an index of resistance to bending, in the plane of the DXA image) are computed directly by the HSA program and require no assumptions about cross-sectional bone shape or proportions of cortical to trabecular bone [27]. To determine average cortical thickness, endosteal diameter and buckling ratio, it is essential to assume a particular shape and cortical/trabecular composition of each HSA ROI [26]. Buckling ratio (an index of wall stability in thin walled tubes) is the ratio of *d*_max_ to average cortical thickness, where *d*_max_ is the larger of the distances between the centroid and either the lateral or medial outer bone edges. The femoral shaft approximates reasonably to circular annuli and contains only cortical bone of minimal porosity (about 5%) in younger women [28]. There are considerable uncertainties about the shape and cortical/trabecular composition of the narrow neck and intertrochanteric regions. Also the cortical/trabecular ratio may conceivably change during a longitudinal study. Hence this paper only reports measurements of average cortical thickness, endosteal diameter and buckling ratio for the femoral shaft and assumes that cortical porosity of the shaft does not change during lactation.

The height and weight of all women were measured at each visit. Calcium intake was estimated using two methods: Calquest food frequency questionnaire (FFQ) (Calquest; Department of Food and Nutritional Sciences, King's College, London) [29] and a prospective 7-day food diary as described previously [2]. FFQs were completed at each visit by all women. In addition, one prospective 7-day food diary was completed at baseline by 22 of the NPNL women. Forty-five of the lactating women completed the 7-day diary at about 2 months postpartum before they had given any solid foods to their infants. Calcium intake from supplements and medication was included in the totals of both the FFQs and food diaries. Full details of these measurements have been published previously [2].

### Statistics

Statistical analyses were performed using linear model software in DataDesk 6.1.1 (Data Description Inc, Ithaca, NY). Differences between NPNL and lactating women, at the time of their first measurement, were investigated using Student's two-tailed *t*-test. Descriptive statistics are reported as means and standard deviations. Changes over time are reported as means and standard errors. Scheffe's post hoc method was used to reduce effects of multiple testing. All variables, except age, were transformed into natural logarithms to normalize skewness where necessary and to determine proportional (percentage) changes of discrete variables [30].

Independent determinants of changes in HSA variables were explored using multiple regression models. Determinants investigated included mean and changes in weight, and mean and changes in calcium intake using the FFQ data obtained at the different timepoints. In addition, data from individual food diaries, obtained at a single timepoint were used to group women with calcium intakes above and below the median to investigate group differences using conditional regression analysis.

## Results

The characteristics of the 48 lactating women at 2 weeks postpartum and 23 NPNL women at baseline are shown in Table 1. There were no significant differences in weight and height between the two groups but the NPNL women were, on average, younger. BMDa was significantly lower for lactating women at the narrow neck (4.2%) and intertrochanter (5.3%) and these differences remained significant after adjusting for age. There were no significant differences for other hip measurements. There was a wide range in calcium intakes for both NPNL and lactating women but the NPNL women had significantly lower intakes of calcium at baseline compared to the lactating women at 2 months postpartum (prospective food diary data expressed as mean [SD, range]: NPNL women 904 [196, 456–1160] mg/day; lactating women 1254 [416, 637–2280] mg/day).

During the study, lactating women lost significant weight (2 weeks postpartum to peak-lactation −2.79 ± 0.72%, *P* < 0.001; 2 weeks postpartum to post-lactation −5.00 ± 0.83%, *P* < 0.0001). In contrast, NPNL women had no significant decrease in weight (0.51 ± 0.89%).

The percentage changes in HSA measurements for lactating women from 2 weeks postpartum to peak-lactation and 2 weeks postpartum to post-lactation, are shown in Table 2. This table also reports the HSA changes observed for the NPNL women during the study. At peak-lactation, significant decreases in BMDa were observed at all three hip sites. Significant decreases in CSA were also observed at the narrow neck and intertrochanteric region. There were no significant changes in bone width at any site. Section modulus decreased significantly only at intertrochanter. At the femoral shaft, cortical thickness decreased significantly, contributing to an increase in buckling ratio. After correcting for changes in weight during and after lactation, the magnitude of the changes in HSA outcomes decreased and only remained significant for BMDa and CSA at the narrow neck and intertrochanteric region.

At the time the women had stopped lactating for at least 3 months, the HSA measurements were, in general, not significantly different from 2 weeks postpartum. The only exceptions were BMDa and CSA of the femoral shaft that remained about 1% below measurements at 2 weeks postpartum. However, after weight correction, there were no significant HSA differences between 2 weeks postpartum and post-lactation for any measurement. During the study, no statistically significant changes in HSA measurements were observed for NPNL women and correcting for changes in weight had minimal effect on results. Neither mean nor change in calcium intake was a significant predictor of change in any HSA variable, whether FFQ or diary estimate was used or whether dichotomization above or below the median was performed.

## Discussion

This study confirms our previous report for these women, using the DXA manufacturer's software, that demonstrated significant but temporary decreases in bone mineral mass during lactation at different sites within the hip [4]. However, this study extends this earlier work by investigating changes in bone structural geometry, as well as bone mineral mass. Knowledge concerning bone geometry is useful to estimate whether lactation influences bone strength and hence makes women more prone to fragility fracture at the hip during or after lactation. Although rare, fragility fractures have been reported during lactation [11,12] and, as described in the Introduction, retrospective studies investigating the relationship between parity and/or lactation history and fracture risk and bone mineral status are conflicting.

Significant decreases in BMDa and CSA were observed at the narrow neck and intertrochanteric regions; indicative of a decreased ability to resist fractures from axial loading. Direct comparison of bone mineral mass changes at HSA-defined hip sites with conventional DXA sites can only be made for the narrow neck region. The observed decrease in BMDa at the narrow neck of – 2.8% is consistent with previous reports, using DXA manufacturer's software, of –2 to –7% at the femoral neck [2–9]. In contrast, at the femoral shaft decreases in BMDa and CSA were smaller than those observed at other HSA sites, and no significant changes remained after weight correction. The femoral shaft, unlike the narrow neck and intertrochanteric regions, contains only cortical bone in younger women. This finding is compatible with previous observations that have shown that decreases in bone mineral during lactation occur predominantly at sites rich in trabecular bone [4].

Bone strength is determined not only by bone mineral mass but also by bone structural geometry. Cross-sectional moment of inertia, section modulus or, indirectly, bone width describes the distribution of mineral mass from the cross-sectional centroid. The further the mineral mass is located from the centroid, the greater is the bone's ability to resist bending deformation. This study observed no significant changes to bone width for any hip region. Hence any cortical bone loss must have occurred at internal surfaces or by increasing intracortical porosity and not at the periosteal surface. Loss of trabecular bone may be due to thinning of trabeculae. If it is assumed that there are no changes in intracortical porosity, results for the femoral shaft provide further evidence for endosteal resorption, as the cortical thickness decreased significantly and endosteal diameter increased, although not significantly. These findings are in keeping with the proposal that the mechanically inefficient endocortical apposition that occurs during puberty in girls, but not in boys, and acts as a reservoir for the calcium required to support future pregnancies and lactations [31].

During the course of the study, the lactating women lost 5% of their body weight. Changes in body weight can influence the interpretation of skeletal changes because body weight affects DXA measurements of bone mineral status physiologically through the loading effects on the skeleton [32]. Adjusting for weight loss reduced the magnitude of the decreases in most of the HSA variables in lactating women (Table 2). This could be interpreted to indicate that some of the observed changes at the narrow-neck and intertrochanteric region, and all observed HSA changes at the femoral shaft, can be attributed to weight change and not to lactation per se. However, weight change could be acting as a surrogate for other factors. For example, breast milk volume has been identified as a significant predictor of changes in spine bone mineral [2] and production of large volumes of breast milk is also likely to contribute to maternal weight loss. Further work is required to determine exactly how weight and other factors contribute to the observed HSA changes.

This study explored the impact of calcium on the lactation-associated bone changes. Although there was a very wide range in the calcium intake of the women (637–2280 mg/day), women selected their own diet and the majority of women were consuming about 1200 mg of calcium per day, close to or above the intakes that are currently recommended [33]. No relationship between dietary calcium intake determined from either FFQs or 7-day food diaries and changes in hip structural geometry, including BMDa, were found during lactation. This finding is compatible with the growing evidence from DXA measurements that suggest the skeletal response to lactation is independent of maternal calcium intake in healthy well-nourished adult women [2,3,5,6].

There are several limitations to this study. These include study design, choice of measurement timepoints and inherently in the HSA method. No postpartum nonlactating women were included and the relatively small number of lactating women comprising the study were recruited after delivery and not prior to pregnancy. Hence some of the observed changes may reflect postpartum changes unrelated to lactation. Also the total effects of the reproductive cycle (pregnancy plus lactation) on hip structural geometry could not be determined. Decreases in bone mineral and area have been reported to occur during pregnancy [34]. This may partially explain the lower BMD at narrow neck and intertrochanter observed in the lactating women at 2 weeks postpartum compared to the NPNL women.

In addition, the duration of lactation in women in the current study varied widely (3 months to more than 2 years) and DXA measurements obtained at both 3 and 6 months (depending on length of lactation) were pooled and defined as peak-lactation. Presently it is unclear whether cessation of lactation or return of menstruation drives the recovery after lactation. In this study 3 months post-lactation, when all women had resumed menstruation, was chosen as the endpoint. It is possible that recovery from lactation was still occurring for some women.

Although the HSA method extends the information traditionally derived from DXA scans, these scanners were not designed for detailed mapping of the spatial distribution of bone mineral. The precision of HSA outcomes has been reported to be approximately two-fold poorer than conventional DXA measurements of BMDa and bone area [35]. The HSA method is based on a simple biomechanical model that aims to account for bending and compressive loadings on idealised ‘beam’ sections comprising the proximal femur. Bending can only be assessed in the plane of the DXA image. Those outcomes relying on the capacity of the method to distinguish between trabecular and cortical bone, even when restricted to the shaft (as in this study), rely on assumptions concerning the unknown shape of the bone cross-section and the invariance of cortical porosity. Interpretation of all HSA outcomes, other than bone width, must take into consideration that structural geometric variables are highly correlated with conventional BMDa [36]. This limits the capacity of a study to distinguish the independent contributions to bone strength of mineral mass and mineral spatial distribution. In osteoporosis diagnosis, structural geometrical analysis has not been able to predict proximal femoral fractures better than BMDa [37]. Nevertheless, HSA provides insight into the influence on bone mechanical strength arising from changes in bone mineral content and its structural deployment that cannot be assessed by an integral variable such as BMDa alone.

In conclusion, this study has shown that human lactation results in significant but temporary alterations to hip bone structural geometry and bone mineral content. Biomechanically, these alterations translate to the narrow neck becoming more susceptible to axial loading (decrease in narrow neck CSA) and the intertrochanter to both bending and axial loading (decrease in CSA and section modulus). Changes in body weight, but not calcium intake, were associated with these alterations. Overall, lactation-associated changes in bone structural geometry and bone mineral content had minimal short-term impact on compressive (CSA) or bending strength (section modulus) in these well-nourished women because alterations occurred mainly at internal surfaces close to the neutral axis and changes in CSA were small. This study also found no evidence for a detrimental effect on bone mineral content or structural geometry after lactation had ceased and therefore on the inferred indices of compressive and bending strength, at each of the sites examined by HSA. Further research is required to confirm these findings in other lactating populations especially among potentially vulnerable women such as adolescent mothers and women with very low calcium intakes (about 300 mg/day).

## Figures and Tables

**Table 1:** Characteristics of lactating women at 2 weeks postpartum and nonpregnant, nonlactating (NPNL) women at baseline.

	Lactating women (*n* = 48)	NPNL women (*n* = 23)
Age (year)	32.1 ± 4.0	27.4 ± 7.0⁎
Height (cm)	165.1 ± 5.9	164.6 ± 6.2
Weight (kg)	67.5 ± 10.4	64.9 ± 14.9

*HSA outcomes*		
Narrow neck		
BMDa (g/cm^2^)	0.892 ± 0.151	0.930 ± 0.110⁎
CSA (cm^2^)	2.73 ± 0.56	2.75 ± 0.40
Bone width (cm)	3.22 ± 0.42	3.12 ± 0.41
Section modulus (cm^3^)	1.31 ± 0.36	1.34 ± 0.33
Intertrochanter		
BMDa (g/cm^2^)	0.845 ± 0.108	0.891 ± 0.118⁎
CSA (cm^2^)	4.24 ± 0.65	4.45 ± 0.68
Bone width (cm)	5.26 ± 0.32	5.24 ± 0.31
Section modulus (cm^3^)	3.55 ± 0.69	3.70 ± 0.71
Femoral shaft		
BMDa (g/cm^2^)	1.377 ± 0.127	1.404 ± 0.172
CSA (cm^2^)	3.81 ± 0.13	3.84 ± 0.62
Bone width (cm)	2.90 ± 0.21	2.86 ± 0.17
Section modulus (cm^3^)	2.02 ± 0.30	2.00 ± 0.41
Endosteal diameter (cm)	1.89 ± 0.27	1.82 ± 0.20
Av cortical thickness (cm)	0.51 ± 0.06	0.52 ± 0.08
Buckling ratio	2.94 ± 0.45	2.83 ± 0.39

Data are mean ± standard deviation. Av = average.

⁎ NPNL women significantly (*P* < 0.05) different from lactating women, determined by Student's two-tailed *t*-test.

**Table 2:** Percentage changes in HSA outcomes in lactating women from 2 weeks postpartum (PP) and from baseline in nonpregnant, nonlactating (NPNL) women.

	Lactating women				NPNL women	
	2 weeks PP to peak-lactation (*n* = 48) % Change		2 weeks PP to post-lactation (*n* = 46) % Change		Baseline to 1 year (*n* = 23) % Change	
	Unadjusted	Adj for wt	Unadjusted	Adj for wt	Unadjusted	Adj for wt
Narrow neck						
BMDa	− 2.77 ± 0.79***	− 2.12 ± 0.89*	−0.65 ± 0.82	− 0.61 ± 1.12	0.51 ± 1.32	0.56 ± 1.35
CSA	− 3.37 ± 0.91***	− 3.07 ± 1.06**	− 0.41 ± 0.78	− 0.04 ± 1.06	2.26 ± 1.16	2.29 ± 1.20
Bone width	− 0.60 ± 0.90	− 0.94 ± 1.04	0.26 ± 0.63	0.58 ± 0.85	1.75 ± 1.90	1.72 ± 1.96
Section modulus	− 2.42 ± 1.57	− 1.94 ± 1.82	0.50 ± 1.36	1.49 ± 1.84	1.77 ± 1.56	1.87 ± 1.60
Intertrochanteric region						
BMDa	−3.18 ± 0.54***	− 2.95 ± 0.63***	− 0.15 ± 0.55	− 0.04 ± 0.75	− 0.08 ± 0.58	− 0.15 ± 0.58
CSA	− 2.73 ± 0.54***	− 2.33 ± 0.61***	0.07 ± 0.55	− 0.06 ± 0.74	0.11 ± 0.66	− 0.02 ± 0.64
Bone width	0.44 ± 0.27	0.62 ± 0.31	0.21 ± 0.39	− 0.03 ± 0.52	0.22 ± 0.47	0.16 ± 0.47
Section modulus	− 2.05 ± 0.83*	− 1.49 ± 0.94	− 0.08 ± 0.79	− 0.09 ± 1.08	− 0.62 ± 0.98	− 0.77 ± 0.97
Femoral shaft						
BMDa	− 1.35 ± 0.59*	− 0.92 ± 0.68	− 1.05 ± 0.52*	− 0.15 ± 0.67	0.82 ± 0.69	0.73 ± 0.70
CSA	− 1.10 ± 0.57	− 0.55 ± 0.64	− 1.24 ± 0.59*	− 0.73 ± 0.79	0.55 ± 0.64	0.42 ± 0.62
Bone width	0.25 ± 0.35	0.37 ± 0.40	− 0.19 ± 0.36	− 0.58 ± 0.48	− 0.27 ± 0.35	0.31 ± 0.35
Section modulus	− 0.82 ± 0.68	− 0.31 ± 0.77	− 1.40 ± 0.82	− 0.97 ± 1.11	0.37 ± 0.89	0.09 ± 0.76
Endosteal diameter	1.24 ± 0.82	1.25 ± 0.95	0.31 ± 0.76	− 0.88 ± 1.00	− 1.08 ± 0.88	− 1.04 ± 0.91
Av cortical thickness	− 1.74 ± 0.79*	− 1.27 ± 0.90	− 1.23 ± 0.67	− 0.04 ± 0.88	1.15 ± 0.92	1.04 ± 0.93
Buckling ratio	2.33 ± 1.00*	2.07 ± 1.15	1.32 ± 0.83	− 0.21 ± 1.08	− 1.41 ± 1.12	− 1.37 ± 1.15

Results are mean percentage change ± SE. Positive values indicate an increase in value, negative a decrease. Unadjusted: percentage change calculated using linear model software with BMDa, CSA, bone width, section modulus, and, in shaft only, endosteal diameter, average (Av) cortical thickness, buckling ratio as the dependent variable and timepoint and subject identity as independent variables. Scheffe's post hoc method was used to reduce the effects of multiple testing. Adj for wt: percentage change after adjustment for weight changes of women. This was determined by constructing multiple regression models and including weight of each woman at each timepoint as an independent variable. Results were determined for full model without backward elimination of nonsignificant variable. Significance of change from first measurement: *P < 0.05, **P < 0.01, ***P < 0.001. Time intervals expressed as mean (SD, range) for lactating women at 2 weeks postpartum to peak-lactation = 142 (41, 64–210) days and 2 weeks postpartum to post-lactation = 411 (131, 255–909) days and for NPNL women at baseline to 1 year = 319 (67, 152–406).
